# The Predictive Value of Amplitude-Integrated Electroencephalography for the Neurodevelopmental Outcomes of Preterm Newborns at 12 Months Corrected Age

**DOI:** 10.3390/children11080979

**Published:** 2024-08-13

**Authors:** Kristina Štuikienė, Elke Griesmaier, Ilona Aldakauskienė, Jurgita Garčinskienė, Marija Paškauskė, Kastytis Šmigelskas, Inesa Rimdeikienė, Vitalija Marmienė, Rasa Tamelienė

**Affiliations:** 1 Department of Neonatology, Lithuanian University of Health Sciences, 44307 Kaunas, Lithuania; 2 Department of Pediatrics II, Medical University of Innsbruck, 6020 Innsbruck, Austria; 3 Faculty of Public Health, Health Research Institute, Lithuanian University of Health Sciences, 44307 Kaunas, Lithuania; 4 Department of Rehabilitation, Lithuanian University of Health Sciences, 44307 Kaunas, Lithuania; 5 Department of Psychiatry, Lithuanian University of Health Sciences, 50161 Kaunas, Lithuania

**Keywords:** amplitude-integrated EEG, premature infant, Burdjalov score, neurodevelopment of premature infant

## Abstract

Background. In clinical practice, it is crucial to identify diagnostic methods that can forecast the neurodevelopmental outcomes of very preterm neonates. Our study aimed to assess the predictive significance of amplitude-integrated electroencephalography (aEEG) for the neurodevelopmental outcomes of preterm infants at 12 months corrected age and to establish the cut-off score that could indicate potential neurodevelopmental impairments. Methods. Preterm neonates born before 32 weeks of gestational age between June 2020 and July 2022 were included in a prospective manner. Amplitude-integrated electroencephalography recordings were conducted at five age intervals (days 1–3; first, second, third and fourth weeks). Recordings were analyzed using the Burdjalov scoring system. The neurodevelopment assessment with Bayley Scales of Infant Development—Second Edition was carried out at 12 months corrected age. Results. A total of 140 newborns were included in the study. Neurodevelopment was assessed in 108 infants at 12 months corrected age. Higher total aEEG Burdjalov scores were observed in groups with normal cognitive and motor development. The most sensitive and specific score for prediction of cognitive impairment in 12 months corrected age was an aEEG evaluation of 5.5 according to Burdjalov score within the first three days. The most sensitive and specific score for prediction of motor impairment was 8.5 within the first week. Conclusions. According to our research there is currently not enough data to accurately foresee the development of newborns at 12 months corrected age according to early aEEG test results. However, conducting a research with bigger sample size and repeated evaluations at a later age might increase the prognostic value of aEEG. In this study cut-off scores of aEEG performed early in life to predict later neurodevelopment outcomes were determined.

## 1. Introduction

Over recent decades, the global number of preterm births has been increasing. According to data from 2020, −13.4 million preterm neonates were born, which made up −9.9% of all neonates in the world that year [1]. As medicine improves, so does the survival probability for very preterm infants. The survival rate for neonates of lower than 28 weeks gestational age is 95% [2,3]. Although these achievements offer some hope, researchers have found that very low birth weight (VLBW) newborns (<1500 g) still face an increased risk of neurodevelopmental impairments [4,5,6,7]. Up to 15% of surviving very preterm newborns experience neurobehavioral impairments, including cerebral palsy and severe neurosensory disorder. About 50–70% of VLBW preterm newborns are observed to have cognitive, behavioral, and social impairments at a later age in life, which hinder adaptation in school and during adolescence and adulthood [2,8,9,10]. Timely identification and management of cerebral damage offers the possibility to prevent poor long-term outcomes. It is crucial to detect these impairments early and apply preventative programs to maximize the long-term health outcomes for very premature newborns. Various technologies are employed to detect brain damage, such as neurosonography and magnetic resonance imaging. These diagnostic measures provide the ability to determine structural brain damage. Over recent decades, more significance has been placed on an electrophysiological assay called amplitude-integrated electroencephalography (aEEG) [11,12,13,14]. The aEEG is becoming increasingly more relevant and is applied more often for very preterm newborns [12,15,16,17]. Research is being conducted all over the world to investigate another purpose of aEEG—whether with the help of aEEG registered in the first hours, days, and weeks of a preterm newborn’s life it is possible to predict the severity of neurodevelopmental impairments experienced during infancy [18,19,20,21,22]. Since research into this topic remains limited, our study aimed to find out the predictive value of aEEG using the Burdjalov scoring system for the neurodevelopmental outcomes of preterm newborns at 12 months corrected age. It was also aimed to determine the cut-off score from which it would be possible to predict neurodevelopmental impairments.

## 2. Materials and Methods

Our study was conducted prospectively at the Department of Neonatology, Hospital of Lithuanian University of Health Sciences Kaunas Clinics, from 1 June 2020, to 30 July 2022.

The study protocol was approved by the Kaunas Regional Biomedical Research Ethics Committee, with permit number BE-2-80 issued on 3 October 2019. Additionally, the study was listed in the ENCPP database under the identifier EUPAS35709.

### 2.1. Study Population

Premature infants who were born and treated at the Department of Neonatology, Hospital of Lithuanian University of Health Sciences Kaunas Clinics, were included in this research.

The inclusion criteria during the neonatal period were these: preterm newborns born before 32 weeks of gestation who had written consent for participation from both parents. Exclusion criteria were multiple developmental anomalies, chromosomal abnormalities, metabolic diseases resulting from genetic disorders, progressive posthemorrhagic hydrocephalus, meningitis, or death before the study was completed.

### 2.2. aEEG Monitoring

Repeated aEEG recordings were performed throughout the first four weeks of life for the enrolled patients. Each amplitude-integrated electroencephalogram was recorded for at least six hours at five designated time intervals: days 1–3 (first recording), days 6–8 (second recording), days 13–15 (third recording), days 20–22 (fourth recording), and days 27–29 (fifth recording) of postnatal age. Postnatal age refers to the chronological age, or the time elapsed since birth.

In this study, the Olympic Brainz Monitor (CE0086, BrainZ Instruments, Natus Medical Incorporated, Oakville, ON, Canada) was used. This two-channel bedside aEEG monitor displays both amplitude-compressed and raw recordings for each hemisphere. Cup electrodes were positioned in the C3, P3, C4, and P4 regions for each recording by a qualified and experienced researcher (certificates Nos. 6383-9531-3706 and 2179-2204-3706). Cross-cerebral aEEG recordings from P3 and P4 were analyzed.

Continuous cerebral activity data were recorded and saved using the Olympic Brainz Monitor, and subsequently analyzed with the Olympic Brainz Viewer software (version OBM00001). Continuous measurement of electrode impedance was used for monitoring the quality of the recordings. Recordings with an impedance greater than 10 kOhms or those exhibiting significant artifacts were excluded from the assessment.

### 2.3. aEEG Analysis

Firstly, the longest continuous segment of minimum 6 h from each recording was selected for evaluation. The aEEG recordings were analyzed using the Burdjalov scoring system, which can be used to assess brain maturity in neonates [23]. The evaluation comprised four components: the background pattern, the beginning and appearance of cyclicity, the lower amplitude border, and the bandwidth span. Bandwidth refers to the voltage span (peak-to-trough) of the tracing and the magnitude of aEEG depression (amplitude of the lower border); the span is the narrowest range between the minimum and maximum voltage margins of the tracing (Table 1). Each parameter was evaluated, and the individual scores were summed to find the overall score for every recording, with a maximum possible total score of 13.

At the time of performing the aEEG recordings, the subjects were not medically anesthetized or sedated and were not diagnosed with acute necrotizing enterocolitis (NEC) or sepsis. These conditions may distort the aEEG readings [24,25,26,27,28].

All recordings were evaluated by the same aEEG specialist (K.Š.*).

### 2.4. Assessment of Neurodevelopment

The neurodevelopment assessment was performed by two trained specialists (V.M., I.R.) at 12 months corrected age using the Bayley scales of Infant Development, Second Edition (Bayley-II) [29]. The assessment consisted of the cognitive (Mental Development Index (MDI)) and motor (Psychomotor development Index (PDI)) scores. Scores were evaluated as normal if they fell within 1 standard deviation (SD) of the mean (e.g., with a mean of 100 and SD of 15, the development index is considered normal when the score is above 85). Mild developmental delay was diagnosed with scores between −1 and −2 SD (greater than 70 and less than 85), moderate delay with scores between −2 and −3 SD (greater than 55 and less than 70), and severe delay with scores more than 3 SD below the mean (below 55).

**Figure 1:**
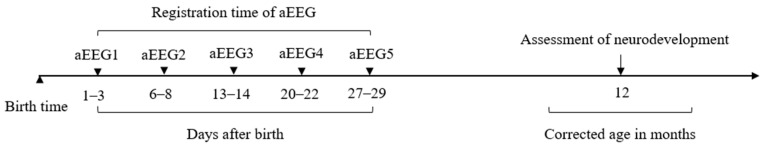
The study process.

Corrected age was calculated by subtracting the number of weeks the neonate was born before reaching 40 weeks of gestation from their chronological age. The entire study process is illustrated in Figure 1.

### 2.5. Collection of Clinical Data

Detailed characteristics of the neonates were acquired from medical records. Early-onset sepsis (occurring less than 72 h after birth) and late-onset sepsis (occurring after more than 72 h) were diagnosed based on clinical signs of generalized infection and abnormalities in laboratory tests. NEC was classified using Bell’s criteria [30], while bronchopulmonary dysplasia was characterized by the need for oxygen supplementation until the 28th day of life, along with an evaluation of need for respiratory support at 36 weeks of postmenstrual age [31]. Hemodynamic significance of patent ductus arteriosus (PDA) was confirmed by echocardiography. Gestational age (in weeks) was calculated as the time from the first day of the last menstrual period to the day of delivery. Progressive posthemorrhagic hydrocephalus was determined as diagnosis of hydrocephalus following intraventricular hemorrhage in premature infants.

### 2.6. Statistical Analysis

The data processing was conducted using SPSS software version 29.0 (IBM Corp. Released 2023, IBM SPSS Statistics for Windows, Version 29.0.2.0, Armonk, NY, USA: IBM Corp). A statistical significance level of *p* < 0.05 was used. Descriptive statistics were estimated with means and medians for central tendency, and standard deviations and interquartile ranges for the dispersion of continuous variables. Categorical variables were presented as absolute numbers (n) and percentages (%). Due to the non-parametric distribution of data in one group, comparisons of Bayley-II scores between two infant groups were performed using the non-parametric Mann-Whitney U test. Burdjalov scores were converted to ranks and their means were compared between the groups.

The Burdjalov aEEG score was considered as the diagnostic test for which the accuracy of results in prediction of impaired development was calculated. This involved assessing the predictive potential (accuracy) of various cut-off points using sensitivity, specificity, positive predictive value (PPV), negative predictive value (NPV), and overall precision, all expressed as percentages. The presence or absence of impaired development was considered as a “Gold Standard” [32].

## 3. Results

### 3.1. Study Population

During time of the study, 230 eligible infants were born and taken care of at the Department of Neonatology, Hospital of Lithuanian University of Health Sciences Kaunas Clinics, a tertiary care facility covering 3/5 of Lithuania. Of these infants, 140 newborns were involved in the study.

aEEG registrations were completed for 123 of these infants, and later assessment at 12 months corrected age was completed for 108 newborns.

**Figure 2:**
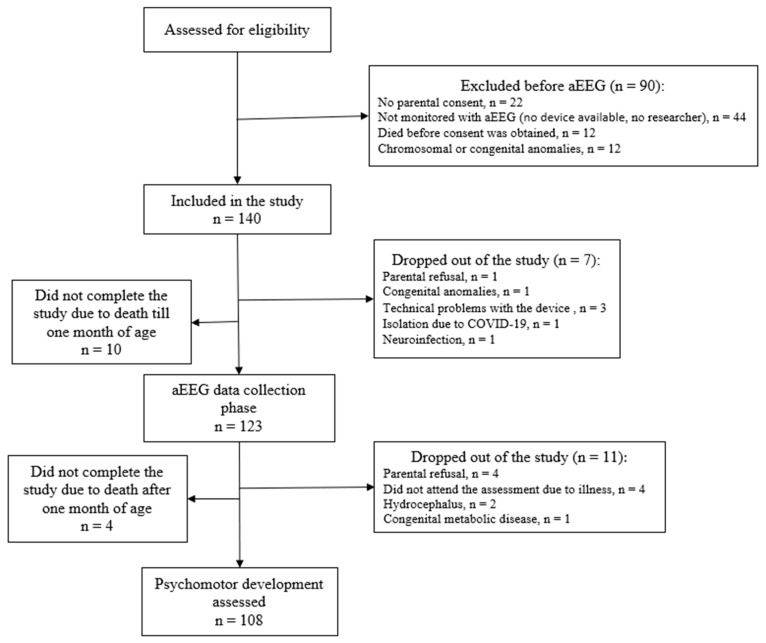
Flow diagram of the study subjects.

A flow diagram of the study subjects’ inclusion and exclusion is shown in Figure 2.

All main characteristics of the subjects in the groups are provided in Table 2. The distribution of variables was very similar between the groups, except morbidities like IVH, PDA and BPD. There were no cases of early onset sepsis among the subjects.

The average (SD) start time of the first aEEG recording was 41 (16) hours after birth.

### 3.2. Predictive Value of the Burdjalov Total Scores

After the assessment of psychomotor development only mild developmental delay was diagnosed.

The individuals were sorted into groups with normal (scores ≥ 85) and delayed (scores ≥70 and <85) neurodevelopment. The Burdjalov aEEG total score results were compared between groups with normal cognitive and delayed cognitive (MDI) development and between groups with normal motor (PDI) and delayed motor development. Cognitive development was assessed for 107 subjects and 108 infants were evaluated for motor development. The distribution of the subjects between the normal and delayed development groups is presented in Table 3.

**Figure 3:**
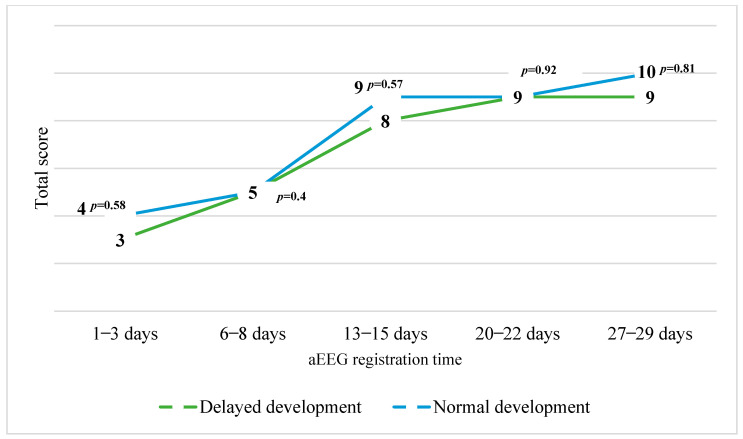
The distribution of the medians of the total Burdjalov scores between the groups with normal cognitive development (MDI) and delayed cognitive development.

After comparing the medians of the total Burdjalov scores between groups with normal and delayed cognitive development, it was determined that all medians of the total aEEG scores were greater in the group with normal cognitive development except at first and third week of age. The differences lacked statistical significance. The distribution of the medians of the total Burdjalov aEEG scores between the groups is presented in Figure 3.

**Figure 4:**
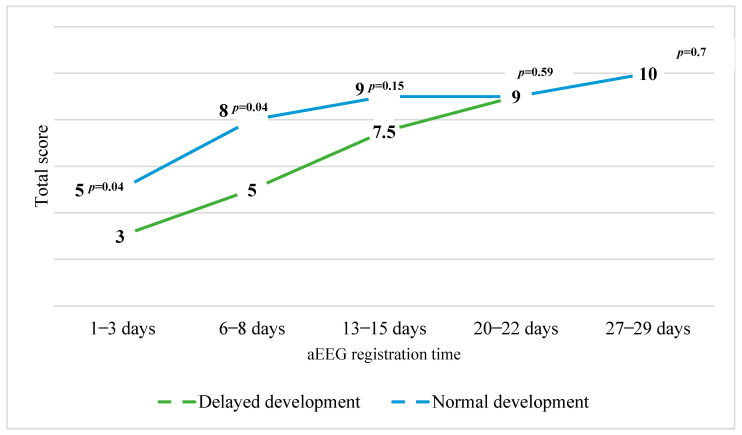
The distribution of the medians of the total Burdjalov scores between the groups with normal motor development (PDI) and delayed motor development.

After comparing the medians of the total Burdjalov scores between groups with normal and delayed motor development, it was determined that all medians of the total Burdjalov aEEG scores were greater in the group with normal motor development. The difference between the medians of the Burdjalov aEEG scores registered during the first three days and week of life was statistically significant. The distribution of the medians of the total Burdjalov aEEG scores between the groups is presented in Figure 4.

Once the Burdjalov scores were calculated, cut-off scores were determined. We were unable to find any data that would allow us to establish cut-off values with both sufficient sensitivity and specificity. Values with the highest sensitivity were selected. The aim was to include as many potential preterm newborns who are likely to experience developmental impairments at 12 months corrected age as possible. After evaluating cognitive development, it was determined that the threshold for the overall Burdjalov score of aEEGs registered in the first three days was 5.5 points with a sensitivity of 86%. As such, if an aEEG total score of a newborn was 6 or more, he or she would be unlikely to experience impaired cognitive development at 12 months corrected age. Specificity for this was 24%, positive predictive value was found to be 22%, negative predictive value was 88%, and precision was estimated to be 36%. The cut-off score for the second aEEG (at 1 week of age) was 9.5 points with a sensitivity of 100%, specificity of 2%, positive predictive value of 20%, negative predictive value of 100%, and precision of 21%. For the third aEEG (at 2 weeks of age), it was calculated to be 9.5 points with a sensitivity of 81%, specificity of 20%, positive predictive value of 20%, negative predictive value of 81%, and precision of 32%. For the fourth aEEG (at 3 weeks of age), it was calculated to be 10.5 points with a sensitivity of 100%, specificity of 8%, positive predictive value of 21%, negative predictive value of 100%, and precision of 26%. For the fifth aEEG (at 4 weeks of age), the cut-off score was calculated to be 10.5 points with a sensitivity of 81%, specificity of 24%, positive predictive value of 21%, negative predictive value of 84%, and precision of 36%.

**Figure 5:**
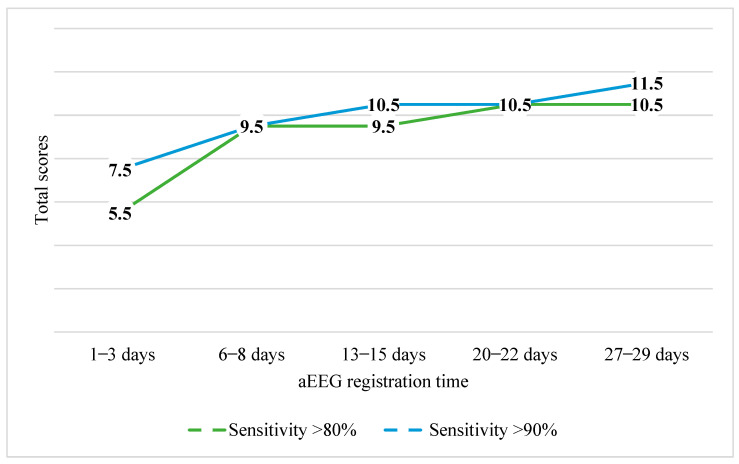
Sensitivity of cut-off total scores in cognitive (MDI) development group.

Figure 5 presents the cut-off scores for cognitive development with sensitivities of >80% and >90%. As demonstrated, with a sensitivity of >90% the scores ranged from 7.5 to 11.5 points.

After evaluating motor development, it was determined that the total Burdjalov cut-off score for aEEGs registered in the first three days was also 5.5 points with a sensitivity of 85%. If the newborn’s aEEG was to receive a score of 6 or more, he or she would be unlikely to experience impaired motor development at 12 months corrected age. Specificity was 33%, positive predictive value 63%, negative predictive value 63%, and precision 63%. The cut-off score for predicting the motor impairment from the second aEEG (at 1 week of age) was 8.5 points with a sensitivity of 84%, specificity of 67%, positive predictive value of 34%, negative predictive value of 63%, and precision of 64%. For the third aEEG (at 2 weeks of age), it was calculated to be 9.5 points with a sensitivity of 84%, specificity of 24%, positive predictive value of 60%, negative predictive value of 52%, and precision of 58%. For the fourth aEEG (at 3 weeks of age), the cut-off Burdjalov score was calculated to be 10.5 points with a sensitivity of 94%, specificity of 7%, positive predictive value of 57%, negative predictive value of 43%, and precision of 56%. For the fifth aEEG (at 4 weeks of age), it was calculated to be 10.5 points with a sensitivity of 98%, specificity of 2%, positive predictive value of 58%, negative predictive value of 50%, and precision of 57%.

**Figure 6:**
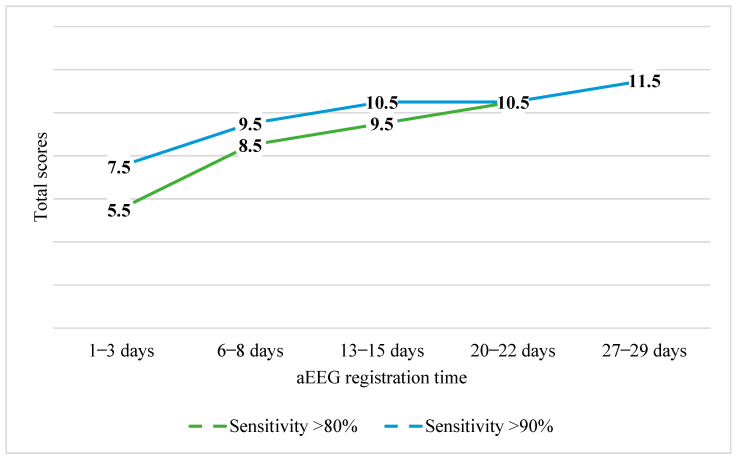
Sensitivity of cut-off total scores in motor (PDI) development group.

Figure 6 presents the cut-off scores for motor development with sensitivities of >80% and >90%. As demonstrated, the scores were mostly higher with a sensitivity of >90%, ranging from 7.5 to 11.5 points.

## 4. Discussion

In modern medicine, the long-term development of preterm newborns is highly important. Knowledge about the possibility of developmental impairments allows neonatologists, pediatricians, and general practitioners to anticipate and start applying early rehabilitation measures. One of the tools for predicting developmental impairments early is the electrophysiological assay aEEG.

Our study aimed to define the predictive value of amplitude-integrated electroencephalography for the psychomotor development of premature newborns at 12 months corrected age.

The study involved even newborns of 23 weeks gestational age. To our knowledge, there is no study that evaluates the aEEG of newborns of such a small gestation and compares it with long-term outcomes at 12 months corrected age.

A similar distribution was observed when comparing characteristics between groups. IVH and PDA were found to be more common in both developmental delay groups. More cases of BPD were found only in the group with delay of motor development.

During our study, we did not observe any severe neurodevelopmental impairments in any patients we assessed at 12 months corrected age. Both motor and cognitive development were good or slightly impaired. Cognitive development at 12 months corrected age was slightly impaired for only 19.6% of the subjects and good for everyone else. After comparing the medians of the total Burdjalov scores between the groups, lower medians were established for all aEEG total scores among the subjects with delayed cognitive development, except at first and third week of age. However, the difference was not determined to be statistically significant. The results were slightly different in motor development evaluations. Mild developmental impairment was found to be present in 57.4% of the subjects. After comparing the medians of the total Burdjalov scores for these subjects, it was found that the scores were statistically significantly lower in the periods of the first three days and one week of age.

When intending to compare the tendencies observed in our study with the results of other studies, we encountered differences in methodology. The study of Welch et al. [33] is based on similar methodological principles, but development was assessed at 18–22 months corrected age. Studies conducted by Kidokoro [34] and El-Dib [35] employed different methodologies for aEEG evaluation. The authors investigated only cyclicity, the sample sizes were very small, and development was evaluated at 12–18 months corrected age. In spite of the differences of methodology, these studies, like ours, did not establish the predictive value of aEEG for the development of premature newborns.

However, there are studies that present data, according to which it is possible to use the results of aEEG evaluations to predict the long-term outcomes of preterm newborns. In a study conducted by Song et al. [22], only severely (MDI <70) impaired cognitive development was evaluated at 18 months corrected age. The study determined a link between Burdjalov aEEG scores and impaired cognitive development. Ralser et al. [21] established that aEEG evaluation scores were statistically different between groups with normal development, light developmental impairments (>70 and <85), and severe developmental impairments (<70). However, the results differed more greatly between groups with normal development and severe developmental impairments.

We were able to discover only a few studies that investigate neurodevelopment at 12 months corrected age with most performing evaluations of neurodevelopment at 24 months corrected age and later [36,37,38,39,40,41]. It may be concluded that a later evaluation determines more pronounced neurodevelopmental impairments and a greater predictive value of aEEG. Differences may also be more prominent with a greater sample size.

Our study presents more tangible differences between the medians of the total Burdjalov scores of the first two registered aEEGs in the motor development group, these results were statistically significant. During that period, preterm newborns are likely experience great health problems which impact their long-term development.

Consequently, based on Burdjalov aEEG total scores, we calculated cut-off scores which can be used to anticipate neurodevelopmental impairments at 12 months corrected age. When predicting cognitive development impairments, the highest percentage of sensitivity and specificity was observed for the Burdjalov total score of 5.5 point of aEEG registered in the first three days. Newborns with an aEEG score of 6 or more would likely not experience impaired cognitive development in the future. When predicting motor development impairments, the highest percentage of sensitivity and specificity was observed for the Burdjalov aEEG total score of 8.5 points registered in the first week. Subjects with an aEEG score of 9 or more points would likely not experience impaired motor development at 12 months corrected age.

When intending to compare the tendencies observed in our study with the results of other studies, after performing an overview of scientific literature, we were able to discover only one relevant study conducted Ralser et al. [21]. Based on aEEG data registered at 18–24 h of age, they determined a cut-off score of 4.5 points (sensitivity of 67% and specificity of 57%) and, based on aEEG data registered at 30–36 h of age, they determined a cut-off result of 5 points (sensitivity of 68% and specificity of 61%). In our study, the average start time for aEEG registration was 41 (16) h, and the cut-off score for both cognitive and motor development was 5.5 points. However, more studies with greater sample sizes are required before guidelines can be proposed.

### 4.1. Strengths

Our study was performed with the most fragile members of our population, preterm newborns, and included neonates of the lowest gestational age (those with gestational age of 23 weeks). There has been little research conducted to investigate the prognostic value of aEEG for the psychomotor development of premature newborns specifically at 12 months corrected age. As such, our study offers a significant contribution to the general community of neonatologists.

### 4.2. Limitations

Firstly, the range of gestational ages in our sample was wide. What is more, the study’s sample size is relatively small, specifically for the subjects with delayed cognitive development, and was dependent on the number of preterm neonates born and taken care of in the Department of Neonatology of the Hospital of Lithuanian University of Health Sciences during the study period. Our study included cases of light cognitive and motor development impairments, and we were not able to compare the aEEG results with the outcomes of severe neurodevelopmental impairments.

## 5. Conclusions

Based on the results of our study, there is currently not enough data to accurately anticipate the development of newborns at 12 months corrected age according to early aEEG test results. However, there are signs that with a bigger sample size and evaluations at a later age the prognostic value may increase. This study allowed us to determine the aEEG cut-off scores—which certainly provide significant value.

## Figures and Tables

**Table 1:** Burdjalov scoring system [23].

Score	Continuity	Cycling	Amplitude of Lower Border	Bandwidth Span and Amplitude of Lower Border
0	Discontinuous	None	Severely depressed (<3 μV)	Very depressed: low span (≤15 μV) and low voltage (5 μV)
1	Somewhat continuous	Waves first appear	Somewhat depressed (3–5 μV)	Very immature: high (>20 μV) or moderate (15–20 μV) span and low voltage (5 μV)
2	Continuous	Not definite, somewhat cycling	Elevated (>5 μV)	Immature: high span (>20 μV) and high voltage (>5 μV)
3		Definite cycling, butinterrupted		Maturing: moderate span (15–20 μV) and high voltage (>5 μV)
4		Definite cycling, noninterrupted		Mature: low span (<15 μV) and high voltage (>5 μV)
5		Regular and mature cycling		

**Table 2:** The key characteristics of the newborns in groups.

Variable	Normal Cognitive Development n = 86	Delayed Cognitive Development n = 21	Normal Motor Development n = 46	Delayed Motor Development n = 62
Smoking in pregnancy, n (%)	9 (10.5)	5 (23.8)	7 (15.2)	7 (11.3)
Cesarean section, n (%)	41 (47.7)	10 (47.6)	20 (43.5)	31 (50)
Antenatal steroids, n (%)	67 (77.9)	16 (76.2)	35 (76.1)	49 (79)
PROM, n (%)	35 (40.7)	9 (42.9)	16 (34.8)	29 (46.8)
Twins, n (%)	36 (41.9)	9 (42.9)	20 (43.5)	25 (40.3)
Gestational age (weeks), Median (IQR)	29 (26.7–30)	28 (27–30)	29 (26.8–30)	28 (27–30)
Birth weight (grams), Median (IQR)	1139 (897–1504)	1116 (1026–1473)	1323 (903–1520)	1083 (894–1398)
Male, n (%)	44 (51.2)	13 (61.9)	26 (56.5)	31 (50)
APGAR score, Median (IQR):				
1-min	7 (6–8)	8 (6–8)	8 (7–8)	7 (6–8)
5-min	8 (7.5–9)	8 (7–9)	9 (8–9)	8 (7–9)
Surfactant treatment, n (%)	43 (50)	12 (57.1)	23 (50)	33 (53.2)
Morbidity:				
Grade III IVH, n (%)	2 (2.3)	2 (9.5)	1 (2.2)	3 (4.8)
Grade IV IVH, n (%)	3 (3.5)	1 (4.8)	0 (0)	4 (6.5)
Cystic PVL, n (%)	1 (1.2)	2 (9.5)	1 (2.2)	2 (3.2)
Late onset sepsis n (%)	14 (16.3)	6 (28.6)	10 (21.7)	11 (17.7)
NEC, n (%)	19 (22.1)	4 (19)	8 (17.4)	16 (25.8)
PDA, n (%)	26 (30.2)	11 (52.4)	13 (28.3)	25 (40.3)
BPD, n (%)	18 (20.9)	4 (19)	5 (10.9)	18 (29)

IVH—intraventricular hemorrhage; PVL—periventricular leukomalacia; NEC—necrotizing enterocolitis; PDA—patent ductus arteriosus; BPD—bronchopulmonary dysplasia.

**Table 3:** Distribution of subjects between groups with normal and delayed development.

	Normal Development (≥85)	Delayed Development (≥70 and <85)
Cognitive (MDI), n (%)	86 (80.4)	21 (19.6)
Motor (PDI), n (%)	46 (42.6)	62 (57.4)

## Data Availability

The data presented in this study are available on request from the corresponding author. The data are not publicly available due to subject confidentiality.

## Abbreviations

aEEG	amplitude-integrated electroencephalography
BPD	bronchopulmonary dysplasia
NEC	necrotizing enterocolitis
NICU	Neonatal Intensive Care Unit
PDA	patent ductus arteriosus
PNA	postnatal age
PVL	periventricular leukomalacia
VLBW	very low birth weight
